# Steroidogenic capacity of the placenta as a supplemental source of progesterone during pregnancy in domestic cats

**DOI:** 10.1186/1477-7827-10-89

**Published:** 2012-10-30

**Authors:** Marta J Siemieniuch, Ewelina Jursza, Anna Z Szostek, Dariusz J Skarzynski, Alois Boos, Mariusz P Kowalewski

**Affiliations:** 1 Department of Reproductive Immunology and Pathology, Institute of Animal Reproduction and Food Research of the Polish Academy of Sciences, Olsztyn, 10-747, Tuwima-St. 10, Poland; 2 Institute of Veterinary Anatomy, Vetsuisse Faculty, University of Zurich, Winterthurerstrasse 260, CH-8057, Zurich, Switzerland

**Keywords:** Placenta, Progesterone, Cat

## Abstract

**Background:**

Until recently, the corpus luteum (CL) was considered to be the main source of progesterone (P4) during pregnancy in the domestic cat (*Felis catus*). However, other possible sources of P4 have not been ruled out. Although feline placental homogenates were found to be capable of synthesizing P4, expression of the respective steroidogenic enzymes has not been investigated at the molecular level. Therefore, in the present study, expression of the two major factors involved in the synthesis of P4 - 3beta-hydroxysteroid dehydrogenase (3betaHSD) and steroidogenic acute regulatory protein (StAR) - was investigated in the feline CL and placenta during the course of pseudopregnancy and pregnancy.

**Methods:**

The mRNA levels of *StAR* and *3betaHSD* were determined using Real Time PCR and their localizations were determined by immunohistochemistry. Placental P4 concentrations, after ethyl extraction, were measured by EIA.

**Results:**

Luteal *3betaHSD* and *StAR* mRNA levels were strongly time-dependent, peaking during mid-pregnancy. The placental *3betaHSD* mRNA level was significantly upregulated towards the end of pregnancy. In the CL, 3betaHSD and StAR protein were localized in the luteal cells whereas in the placenta they were localized to the maternal decidual cells. Placental P4 concentrations were low in early pregnant queens, but increased along with gestational age.

**Conclusions:**

These results confirm that the placenta is an additional source of P4 in pregnant queens and can thereby be considered as an important endocrine organ supporting feline pregnancy.

## Background

Female domestic cats (*Felis catus*) are traditionally classified as seasonally polyestrous with ovulation provoked by coitus [1,2]. However, in as many as 50% of domestic cats, ovulation occurs without cervical or vaginal stimulation [3,4]. If ovulation is not followed by pregnancy, the queen enters pseudopregnancy during which the corpora lutea (CL) remain active. Hence, the reproductive cycle in the domestic cat differs significantly from that in ungulates [5,6] and dogs [7,8], in which spontaneous ovulation is followed by the formation of CL in each estrous cycle. In the cat [9], rabbit [10] and rat [11], the luteal phase of non-pregnant animals lasts only one-half of a normal gestation. This means elevated progesterone (P_4_) levels measured in circulating blood for 35–40 days in pseudopregnant and for approximately 60–65 days in pregnant queens. In contrast, in the dog [12], mink [13] and ferret [14], the length of the luteal phase is similar in the presence or absence of pregnancy. Progesterone levels in pregnant and non-pregnant dogs are similar within the first 60 days after ovulation [15]. Moreover, there are no indications of an active luteolytic principle in non-pregnant dogs, and, the canine CL appears to be devoid of PGF_2α_-synthase (PGFS) activity [16,17]. Consequently, luteal regression in non-pregnant bitches seems to be a passive degenerative process in the absence of endogenous PGF_2α_[16,17]. The domestic cat thus seems to have a reproductive advantage over some other carnivores because its shorter luteal phase allows for an earlier return to estrual cyclicity. In the cat, ovarian activity returns within 7 to 10 days following pseudopregnancy [10]. The histological and endocrine patterns of early CL formation are the same in pregnant and pseudopregnant cats until days 10–12 after coitus [18]. However, after days 12–13, which coincides with the time of implantation [19,20], plasma P_4_ concentration slowly declines in pseudopregnant queens, whereas during pregnancy it remains on a plateau until day 30 and then gradually declines towards the end of gestation [21].

Until the 1970s, the CL was considered to be the main source of P_4_ during feline gestation. However, the finding that ovariectomy in queens after day 45 either did not interrupt pregnancy [22] or only did in some animals [23] supported an assumption that the placenta is a source of steroidogenic enzymes [18]. Subsequently, feline placental homogenates were found to be capable of synthesizing P_4_[19]; however, the expression of steroidogenic factors or P_4_ content has not been examined in the placenta to date.

Cholesterol is the precursor of all steroid hormones. Steroidogenesis is regulated by steroidogenic acute regulatory (StAR) protein, which controls cholesterol transport from the outer to the inner mitochondrial membranes of the steroidogenic cells [24]. Conversion of pregnenolone to P_4_ is a step catalyzed by 3β- hydroxysteroid dehydrogenase/isomerase (3βHSD). In this study, expression of these two factors was investigated in the feline CL and placenta throughout pregnancy and pseudopregnancy.

## Methods

### Animals and tissue preservation

All procedures were approved by the Local Animal Care and Use Committee in Olsztyn, Poland (No. 41/2007/N and 61/2010/DTN).

Privately owned queens (n = 36, mixed breed, proven health status), aged 18 ± 5 months, housed individually or in pairs, with or without contact with an intact male, were enrolled in this study. No pharmacological treatment was performed to provoke ovulation in the animals. In the case of pregnant queens, an ovulation was provoked with coitus. Queens were checked daily for behavioral signs of estrus (treading of the hind feet, lordosis and tail deflection). The first day of silencing of estrus behavior in the queens was considered as Day 1 of the luteal phase. Reproductive tracts were collected from 13 pseudopregnant cats at early (n = 4, days 3–5), mid (n = 4, days 10–15) and late phases of pseudopregnancy (n = 5, days 25–35), and from 12 pregnant cats at early (n = 5), mid (n = 4) and late (n = 3) gestation: 1.5-2, 3–4 and 6–8 weeks after mating, respectively. For extraction of P_4_ from placentas, an additional 11 cats at different gestational ages were subjected to ovariohysterectomy (OHE), which was done with the owners’ request and consent. The stage of the estrous cycle was further confirmed by endocrine analysis, according to the P_4_ values reported in the literature [10,25], and macroscopic observation of the ovaries and uterus. Before surgery, blood samples were collected from the cephalic vein into EDTA-containing tubes (Tyco Healthcare Group LP, Mansfield, USA) and transported to the laboratory at 4°C. Plasma obtained after blood centrifugation (3500 x g, 10 min) was frozen at −20°C until P_4_ measurement using direct enzyme immunoassay (EIA). Confirmation of gestational age was done according to the P_4_ values reported in the literature [18]. In addition, measurements of the crown-rump length in fetuses and uterine ampullae diameter or length were performed [26,27].

Tissues were washed immediately after surgery with sterile saline to remove blood contamination, then placed into fresh sterile saline at 4°C and transported to the laboratory within 1 h. The CL were removed from a randomly selected ovary among the pairs belonging to the same luteal stage. The second ovary was fixed in buffered 4% formaldehyde for 24 h, dehydrated and wax-embedded. Sections (2–3 μm thick) were used for evaluation of protein concentrations by immunohistochemistry. In pregnant animals, uterine horns were slit longitudinally and fragments of placenta were separated, washed in fresh saline to remove blood, preserved overnight at 4°C with RNA*later* (Ambion Biotechnologie GmbH, Wiesbaden, Germany), and then stored at −80°C until total-RNA extraction. mRNA levels were determined using Real Time PCR, while localization of 3βHSD and StAR protein was examined by immunohistochemistry.

### Cloning of feline 3βHSD by reverse transcription (RT) and rapid amplification of cDNA ends polymerase chain reaction (RACE PCR)

Total RNA was isolated from feline CL using Trizol Reagent according to the manufacturer’s instructions (Gibco-BRL, Life Technologies, Karlsruhe, Germany). The whole procedure was carried out as described before for canine 3βHSD [28]. For initial RT-PCR, 0.2 μg of total RNA was used. An alignment of the known canine 3βHSD sequence (GenBank accession number: AY739720) against the available online feline genomic sequence [29] was performed using BLAST® software to obtain feline-specific PCR product (primers 1–2; 1Table 1). Integrity of RNA was checked by amplification of the housekeeping gene β-actin (primers 3–4, Table 1).

**Table 1:** List of primers used for RT-PCR, RACE PCR and Real Time PCR

**Primer no.**	**Gene**	**Primer sequences**	**Accession number**
1.	Initial 3βHSD for RT-PCR	FWD 5′-TTGATACCCCGGTTTGACCA −3′	AY739720
2.	Initial 3βHSD for RT-PCR	REV 5′-TTGGCACCCCTAGATCAGTG −3′	
3.	ACTB for RT-PCR	FWD 5′-ATCAAGGAGAAGCTGTGCTACGT-3′	AB51104
4.	ACTB or RT-PCR	REV 5′-CGTTGCCGATGGTGATCA −3′	
5.	3βHSD RACE PCR GSP	FWD 5′-CACCAAAGCTATGATAACCTCAAT −3′	AY739720
6.	3βHSD RACE PCR GSP	REV 5′-AGGCAAGCCAGTACTCCAGAAAT −3′	
7.	Universal Primer Mix (UPM)	FWD 5′-CTAATACGACTCACTATAGGGCAAGCAG	-
		TGGTATCAACGCAGAGT-3′	
8.	UPM	REV 5′-CTAATACGACTCACTATAGGGC-3′	AY739720
9.	3βHSD for RT-PCR (ORF)	FWD 5′-TTGATACCCCGGTTTGACCA −3′	
10.	3βHSD for RT-PCR (ORF)	REV 5′-TTGGCACCCCTAGATCAGTG −3′	JF794032
11.	3βHSD specific for Real Time PCR	FWD 5′-TCCCCAGTGTTTCTGATTCC −3′	
12.	3βHSD specific for Real Time PCR	REV 5′-CACCAACAAATGCACGATTC −3′	EF522840
13.	Initial StAR for RT-PCR	FWD 5′-CAATGCTCCTAGCGACGTTC −3′	
14.	Initial StAR for RT-PCR	REV 5′-AACAGTTGGAAGCAGCAGG −3′	
15.	StAR for Real Time PCR	FWD 5′-CCCATGGAGAGGCTTTATGA-3′	JF800676
16.	StAR for Real Time PCR	REV 5′-CAACTCGTGGGTGATGACTG −3′	
17.	Cyclophilin for Real Time PCR	FWD 5′-CCTTCTGTAGCTCGGGTGAG −3′	AY029366
18.	Cyclophilin For Real Time PCR	REV 5′-CTTGGAGGGGAGGTAAGGAG −3′	

First strand cDNA synthesis was performed with the PowerScript Reverse Transcriptase kit (BD Biosciences Clontech GmbH, Heidelberg, Germany) using 0.6 μg of total RNA. Subsequently, the SMART RACE cDNA Amplification kit (BD Biosciences Clontech GmbH) was used with gene-specific primers (GSP, primers 5–6; Table 1) in combination with universal primer mix (UPM, primers 7–8; Table 1) supplied by the manufacturer of the SMART RACE Kit. Overlapping products of the missing cDNA coding fragments of the 5^′^ and 3^′^ ends were amplified. After initial denaturation at 94°C for 1 min, the reactions were run for 35 cycles (94°C for 1 min, annealing at 65°C for 2 min, elongation at 72°C for 3 min), and the final extension was at 72°C for 10 min. Finally, RT-PCR for 40 cycles was performed at an annealing temperature of 57°C with specific primers (primers 9–10; Table 1) located at both ends of the open reading frame (ORF). All PCR products were visualized on a 1.5% ethidium bromide-stained gel, purified with a Qiaex II agarose gel extraction kit (Qiagen GmbH, Hilden, Germany), ligated into pGEM-T vector (Promega, Dübendorf, Switzerland), multiplied in XL1 BLUE competent cells (Stratagene, La Jolla, CA, USA) and sequenced (Microsynth, Balgach, Switzerland). Finally, the cloned cDNA sequence was submitted to GenBank with the accession number: JF794032, Felis catus 3β-hydroxysteroid dehydrogenase mRNA, complete cds.

### Cloning of feline StAR cDNA

The procedure leading to characterization of feline StAR protein was carried out as previously described for canine StAR protein [30]. Total RNA was obtained from three feline CLs collected at early, mid and late phases of pseudopregnancy. The DNase- treatment was performed with RQ1 RNase free DNase (Promega), and the RT-PCR was done with the GeneAmp Gold RNA PCR kit (Perkin-Elmer Applied Biosystems GmbH, Weiterstadt, Germany), all as previously described [30]. Primers for qualitative PCR were obtained from the alignment of the canine sequence with GenBank accession number EF522840 using an online available feline genomic sequence. Using primer pairs (13–14; Table 1), a PCR product comprising 886 bp of feline StAR protein was amplified. PCR conditions were as follows: initial denaturation at 95°C for 10 min, then reactions were run for 40 cycles consisting of denaturation at 94°C for 1 min, annealing at 56°C for 1.5 min and elongation at 72°C for 1.5 min; the final extension was at 72°C for 10 min. PCR products were visualized on a 1.5% ethidium bromide-stained gel, purified with the Qiaex II agarose gel extraction kit (Qiagen GmbH, Hilden, Germany), ligated into the pGEM-T vector (Promega) multiplied in XL1 BLUE competent cells (Stratagene, La Jolla, CA, USA) and sequenced (Microsynth, Balgach, Switzerland). The cloned cDNA sequence was submitted to GenBank with the accession number JF800676 (Felis catus Steroidogenic Acute Regulatory protein (StAR) mRNA cds).

### Real Time PCR

The levels of mRNA expression of target genes were examined by Real-Time PCR using specific primers for *3βHSD, StAR* and cyclophilin *(Cyc)*. Among several different genes, including *β-Actin*, *GAPDH* and *Cyc*, the last one was employed as a reference because the differences in *Cyc* expression between different samples did not exceed two cycles. All primers were purchased from Microsynth (Balgach, Switzerland). The forward and reverse sequences used for quantitative Real-Time PCR and the GenBank accession numbers are given in Table 1 (primers 11–12, 15–16 and 17–18). The Real-Time PCR reactions were carried out in an automated fluorometer ABI PRISM® 7300 Sequence Detection System (Applied Biosystems, Darmstadt, Germany) using SYBR Green Master Mix (Applied Biosystems, Applera, Warsaw, Poland). PCR reactions were performed in 96-well plates. The total reaction volume was 20 μl containing: 1 μl cDNA (200 ng), 250 nM each of forward and reverse primers, and 10 μl SYBR Green PCR Master Mix. Real time PCR was carried out as follows: initial denaturation (10 min at 95°C), followed by 40 cycles of denaturation (15 s at 95°C) and annealing (1 min at 60°C). After each PCR reaction, melting curves were obtained by stepwise increases in temperature from 60 to 95°C to ensure single product amplification. The presence of the product was also confirmed by electrophoresis on 2% agarose gel. Relative quantification was performed by normalizing the signals of target genes with the *Cyc* signal by the Miner method for quantifying qRT-PCR results using calculations based on the kinetics of individual PCR reactions [31].

### Immunohistochemistry

Immunohistochemistry was done according to the procedure described by Kowalewski et al. [30]. Firstly, the sections were deparaffinized and rehydrated, and then incubated in citrate buffer (10 nM, pH 6.0) for 15 min under microwave irradiation at 560 W for antigen retrieval. After cooling for 20 min at 20°C, sections were incubated in 0.3% H_2_O_2_ in methanol for 30 min to quench endogenous peroxidase and then washed in IHC-buffer/0.3% Triton X pH 7.2-7.4 (0.8 mM Na_2_HPO_4_, 1.47 mM KH_2_PO_4_, 2.68 mM KCl, 1.37 mM NaCl). To block nonspecific binding sites, sections were incubated in 10% goat or horse serum. The first primary antibody (dilution 1:5000) was a rabbit polyclonal antiserum against human placental 3βHSD that was kindly donated by Dr I.J. Mason (Clinical Biochemistry, Centre for Reproductive Biology, University of Edinburgh) [32]. The second primary antibody (dilution 1:3000) was a rabbit polyclonal antibody against StAR protein. This was an antipeptide antiserum against amino acids 88–98 of mouse StAR protein kindly donated by Dr. Douglas M. Stocco (Texas Tech University Health Sciences Center, Lubbock, US) [33]. Serum from a non-immunized rabbit served as an isotype control. The third primary antibody (dilution 1:100) was a mouse monoclonal anti-vimentin clone 3B4 (DakoCytomation, Glostrup, Denmark). Vimentin was used to identify cells of mesenchymal origin (*e.g.*, maternal decidual cells, fibroblasts or blood vessels) and distinguish them from non-mesenchymal cells such as fetal trophoblast cells, in the placenta [34]. Sections were incubated overnight at 4°C, then washed in IHC-buffer and incubated with biotinylated goat IgG (secondary antibody, dilution 1:100) against rabbit immunoglobulin (Vector Laboratories, Burlingame, US) (used as a secondary antibody for the sections stained against StAR and 3βHSD) or biotinylated horse IgG (secondary antibody, dilution 1:200) against mouse immunoglobulin (Vector Laboratories, Burlingame, US) (used as a secondary antibody for the sections stained against vimentin). Then the sections were incubated with the avidin-biotin-peroxidase complex (Vectastain ABC kit, Vector Laboratories, Burlingame, US) for 30 min at 20°C. After washing with IHC-buffer, sections were allowed to react with the substrate diamino-benzidine (DAB; DakoCytomation, Glostrup, Denmark) according to the manufacturer’s instructions. The reactions were arrested by dipping the sections under running tap water for 5 min. Hematoxylin was used to counterstain the sections, then they were mounted in Histokit (Assistant, Osterode, Germany).

Isotype control was done to avoid false positive results. The luteal and placental sections were incubated with serial dilutions of pre-immunized rabbit serum (starting at 1:1000) or pre-immunized mouse serum (starting at 1:50). No positive staining was observed at a 1:3000 dilution of pre-immunized rabbit serum or at a 1:100 dilution of pre-immunized mouse serum.

### Placental progesterone extraction

Progesterone extraction was carried out using a modification of a methoddescribed previously [35]. Placenta samples were stored at −80°C. The tissues (200–300 mg each) were thawed and homogenized in glass vials using a tissue disruptor with 400 μl EIA buffer containing 45 μl 1 N HCl. Next, 3 ml of ethyl petroleum was added to each sampleand it was shaken for 10 min, then samples were incubated at −20°C for 4 h. Afterwards, the supernatant was collected and evaporated to dryness under nitrogen at 40°C. Finally, 400 μl of EIA buffer with 0.1% BSA was added. The suspension was frozen at −20°C until P_4_ measurement by EIA.

### Progesterone determination

Assessment of P_4_ concentration in plasma followed the methodology previously described [36]. Horseradish peroxidase-labeled P_4_ was used at a final dilution of 1:75.000. Anti-P_4_ serum (final dilution of 1:100 000) was kindly donated by Dr. Stanisław Okrasa, University of Warmia and Mazury, Olsztyn, Poland. The ED_50_ for P_4_ was 4.2 ng/mL and the assay ranged from 0.39 to 100 ng/mL. The intra- and inter-assay CVs were 4.2% and 9.3%, respectively. Progesterone concentration in plasma was expressed as ng per mL (ng/mL) and in placenta as ng per g (ng/g).

### Statistics

To test the effect of different stages of pregnancy or pseudopregnancy on mRNA levels, the Kruskal-Wallis Test (a nonparametric ANOVA) was used followed by the Newman-Keuls Multiple Comparison Test using the statistical software program GraphPad5 (GraphPad PRISM v 5.0; GraphPad Software Inc., San Diego, CA, USA). Progesterone concentrations in plasma are shown as a mean ± standard deviation. To test the effect of the presence or absence of pregnancy on circulating P_4_ concentrations within each luteal phase, a nonparametric ANOVA was used followed by the Newman-Keuls Multiple Comparison Test. Significance was defined as values of *P* < 0.05.

## Results

### Determination of pregnancy or pseudopregnancy stages

**Figure 1:**
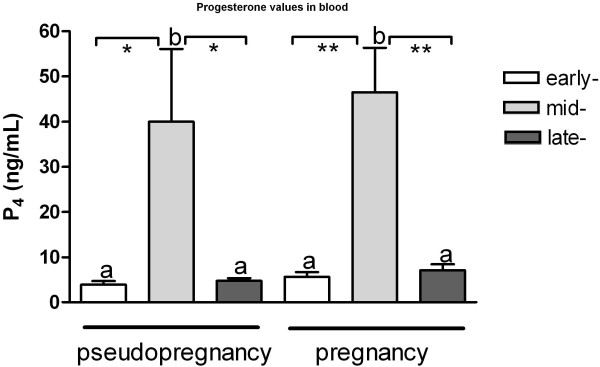
**Progesterone values in blood of queens at early, mid and late pseudopregnancy or early, mid and late pregnancy.** Letters “a, b” indicate statistical differences among groups. Asterisks indicate statistical differences in P_4_ levels between groups within pregnancy or pseudopregnancy conditions (*P < 0.05, **P < 0.01).

**Figure 2:**
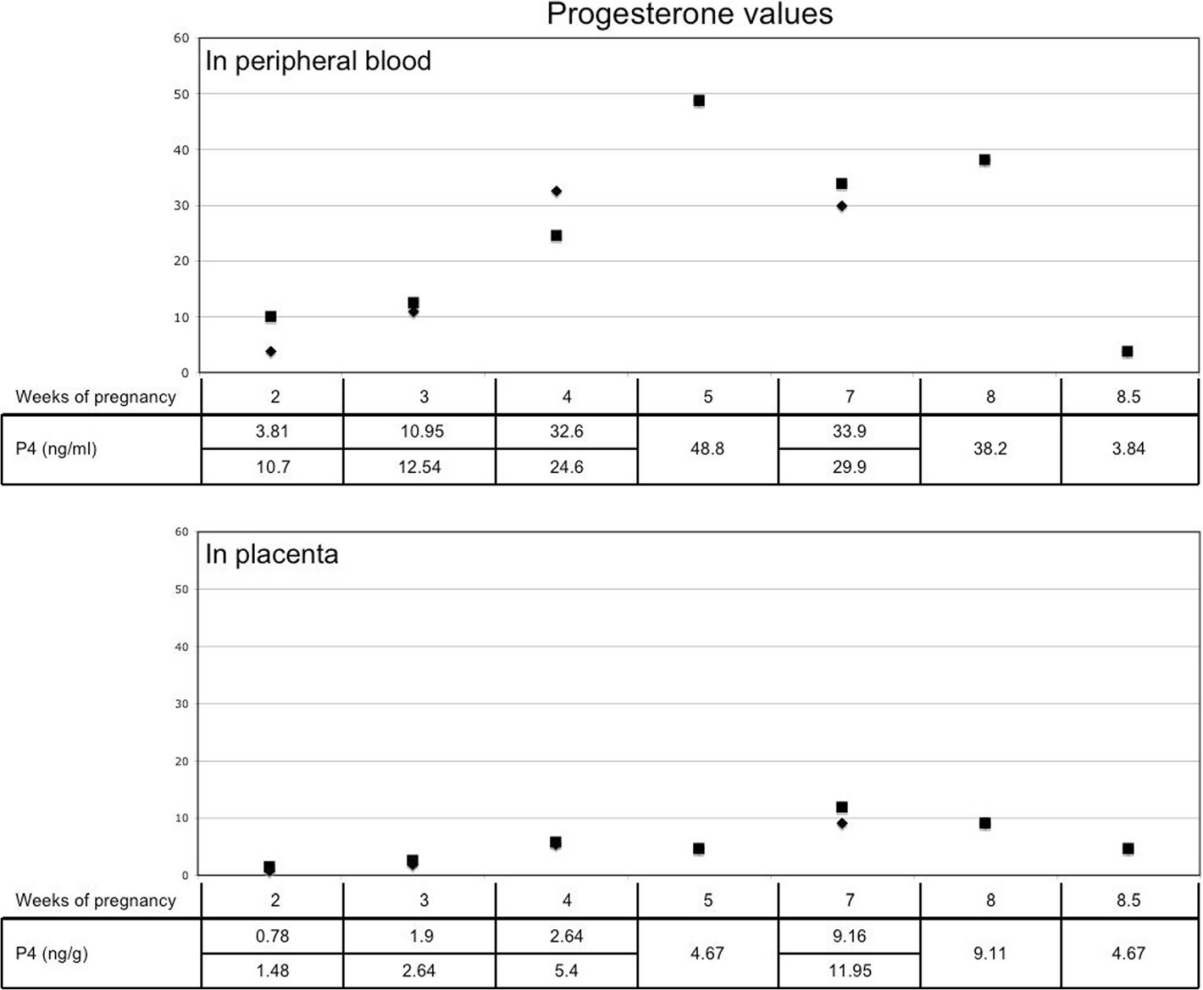
**Progesterone values in blood and placenta.** Progesterone values in blood (upper part) and placenta (lower part) in cats ovariectomized at different weeks of pregnancy.

Queens were considered to be at the early luteal phase when the plasma P_4_ was between 2 to 8 ng/mL and the corpora haemorrhagica were ≥ 2 mm in diameter. Mid-pseudopregnancy was determined when plasma P_4_ was > 17 ng/mL and CL were reddish and 3–4 mm in diameter. Late pseudopregnancy was characterized by plasma P_4_ < 5 ng/mL and the presence of pale CL. The queens were considered to be at early, mid and late pregnancy when the plasma P_4_ was between 2 to 8, > 20 or < 12 ng/mL, respectively. Progesterone concentrations in plasma are shown in Figure 1. The serum P_4_ concentration was 3.9 ± 1.4 ng/mL, 39.9 ± 32.2 ng/mL and 4.2 ± 1.2 ng/mL in queens at early, mid and late pseudopregnancy, respectively; and 5.4 ± 2.3 ng/mL, 46.5 ± 19.6 ng/mL and 5.3 ± 6.4 ng/mL in queens at early, mid and late pregnancy, respectively. No statistical differences were seen within each luteal phase examined with respect to the presence or absence of pregnancy (P > 0.05). Placental P_4_ contents depended on the gestational age and are shown in Figure 2. The amount of extracted P_4_ was 1.7 ± 0.77 ng/g, 5.5 ± 0.58 ng/g and 8.7 ± 3.01 ng/g of tissue from early, mid and late pregnant queens, respectively.

### StAR- and 3βHSD- mRNA levels in the feline CL and placenta

**Figure 3:**
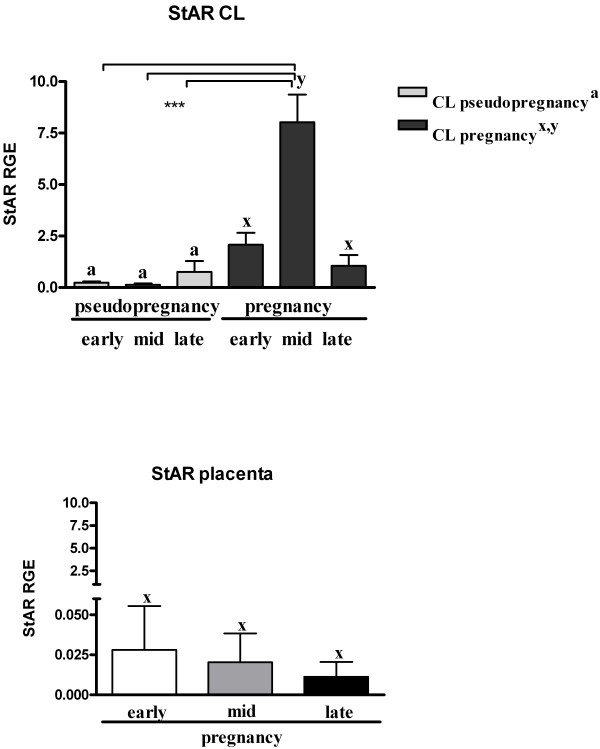
***StAR *mRNA transcription.***StAR* mRNA transcription in pseudopregnant and pregnant CLs (upper panel) and placenta (lower panel). Letter “a” indicates no statistical differences within pseudopregnancy. Letters “x, y” indicate statistical differences within pregnancy (*P* < 0.001). Asterisks indicate statistical differences between *StAR* mRNA levels during the course of pseudopregnancy and pregnancy (***P < 0.001).

Luteal *StAR* mRNA was strongly time-dependent, with significantly elevated mRNA-levels observed during mid-pregnancy (*P* < 0.001) (Figure 3). No statistically significant changes were observed among placental StAR-mRNAs detected at early, mid and late gestation (Figure 3).

**Figure 4:**
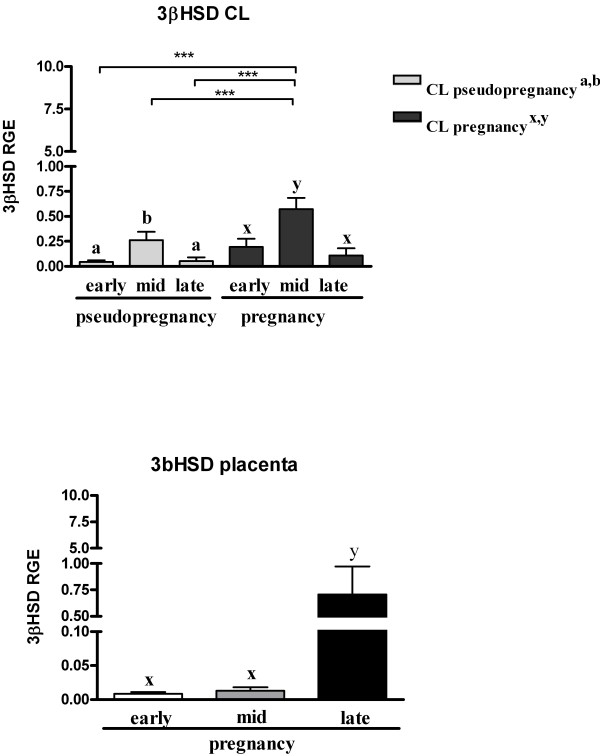
***3β-HSD*-mRNA expression in pseudopregnant and pregnant CLs (upper panel) and placentas (lower panel).** Letters “a, b” indicate statistical differences within pseudopregnancy (*P* < 0.05). Letters “x, y” indicate statistical differences within pregnancy (*P* < 0.01). Asterisks indicate statistical differences between *3β-HSD*-mRNA levels during the course of pseudopregnancy and pregnancy (***P < 0.001).

Luteal *3βHSD* mRNA expression was also strongly time-dependent, with significantly elevated mRNA levels observed during mid-pseudopregnancy (P < 0.05) and mid-pregnancy (P < 0.01) (Figure 4). Placental *3βHSD*-mRNA was significantly up-regulated towards the end of pregnancy (P < 0.01) (Figure 4).

### Localization of StAR protein and 3βHSD in the feline CL and placenta

**Figure 5:**
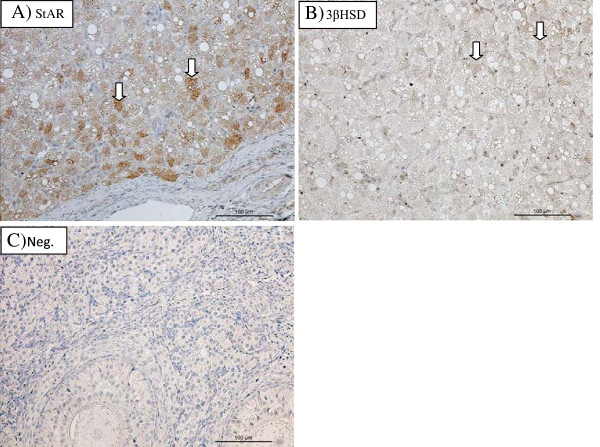
**StAR (A) and 3βHSD (B) protein expression and negative control (C) in CL.** Tissue collected at 8^th^ week of pregnancy. Arrows show luteal cells. Bar represents 100 μm.

**Figure 6:**
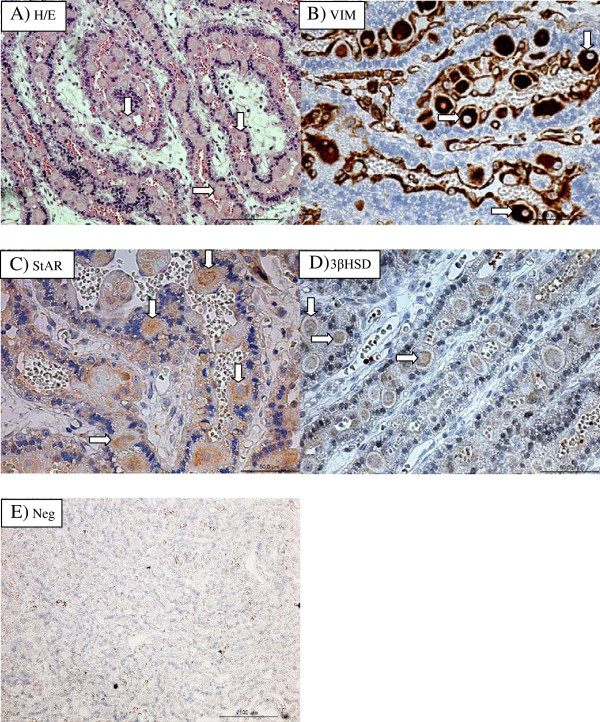
**Hematoxylin/eosin (A) staining and vimentin (B) protein expression, StAR (C) and 3βHSD (D) protein expression and negative control (E) in placentas collected at the 8**^**th**^**week of pregnancy.** Arrows show maternal decidual cells. Bar represents 50 μm in the Figure showing staining for vimentin, StAR and 3βHSD or 100 μm in the Figure showing staining with hematoxylin/eosin and negative control.

The spatial localizations of StAR and 3βHSD proteins in the gestational CL and placenta are shown in Figures 5 and 6, respectively. Within the CL, StAR and 3βHSD protein were localized in the luteal cells (Figure 5A and 5B, respectively). Immunostaining against StAR protein and 3βHSD was observed in early, mid and late pseudopregnancy CL, as well as in early, mid and late pregnancy CL. Based on comparison with the pattern of staining with vimentin (Figure 6B), the placental cells in which StAR (Figure 6C) protein and 3βHSD (Figure 6D) were immunolocalized were identified as cells of mesenchymal origin such as maternal decidual cells (Figure 6B). Positive staining against both proteins examined was generally observed in mid and late pregnancy placentas. A slight positive staining against 3βHSD was also observed in the fetal component of the placentas.

## Discussion

The present results confirm that the placenta is an additional source of P_4_ in pregnant queens, possibly acting as an important endocrine organ during pregnancy. Both StAR and 3βHSD were immunolocalized in the placenta and CL in each luteal phase. In the present study, the luteal *3βHSD* mRNA expression patterns in pregnant and pseudopregnant animals were similar, both peaking during the mid-luteal phase. However, luteal mRNA was 2.5-fold higher in pregnant than in pseudopregnant queens. Luteal *StAR* mRNA remained at a relatively constant low level in pseudopregnant animals, but followed the pattern of *3βHSD* mRNA expression in pregnant animals. However, no quantitative assessment was performed for the expression of StAR and 3βHSD at the protein level.

A rapid development of the P_4_-producing CL is observed in both pregnant and pseudopregnant cats. Plasma P_4_ levels are about the same in pregnant and pseudopregnant queens in the first 10–12 days after coitus [18] but their profiles diverge as early as on day 12 or day 13 after coitus, which is the period of implantation in the cat [18,20]. The luteal *StAR*- and *3βHSD*- mRNA were highest during the mid-phase of pregnancy. Additionally, increased *3βHSD*-levels were also observed at the mid-phase of pseudopregnancy. The high expression of both factors during both mid-luteal phases may be responsible for the strongly elevated P_4_ levels in the circulating blood reported previously [2,18,21,37] and observed also in the present study. As reported previously, P_4_ values rose rapidly in pseudopregnant queens between Day 4 and Day 9, peaking around Day 14 post-ovulation with a plateau between Day 9 and Day 23. The range of serum P_4_ concentrations at that time was 30 ng/mL to more than 87 ng/mL [2]. The present results concur with data reported earlier, in which strong individual variations in plasma P_4_ concentrations were observed. In one queen experiencing a nongravid luteal phase, P_4_ peaked at 88 ng/mL, *vs*. a range of 20.1-28.7 ng/mL observed in other animals. The mean value of plasma P_4_ observed in queens at the mid-luteal stage was 39.9 ng/mL and is consistent with the those reported in the literature [2]. However, in another study, P_4_ values in pseudopregnant cats were shown to peak at levels of 17 ng/mL on Day 18 postcoitum, followed by a gradual decline to values < 1 ng/mL between Days 30–46 after coitus [25]. In pseudopregnant queens at the late luteal stage, we usually observed distinctly diminished P_4_ values compared to the mid luteal phase. Wildt and coworkers [2] reported that the serum P_4_ values were lower than 1 ng/mL by Day 42 post ovulation, however, CL remnants were visually evident throughout the following interestrus period. In the present study, the P_4_ values in plasma reached approximately 4.2 ± 1.2 ng/mL. However, blood samples were collected from pseudopregnant queens only from Day 25 to Day 35 (Day 1 signified the first day of pseudopregnancy and was consistent with silencing of estrus behavior), so this difference might explain the discrepancy in observed P_4_ values.

During mid-pregnancy, the serum P_4_ values varied significantly between individual cats (range 34 – 74.4 ng/mL), with a mean P_4_ concentration of 46.5 ng/mL. Although P_4_ concentrations in the blood of mid-pregnant queens were numerically higher than in non-pregnant animals in the mid-luteal phase, no statistical differences were seen in the present study between these two groups. It cannot be ruled out that these results might differ significantly if the number of females in each group were increased; however, it should also be mentioned that the present data are very similar to those reported by others (for review see: 2, 37). Distinct individual variations in the plasma P_4_ levels collected from pregnant cats were previously reported [37], *i.e*., peak serum P_4_ from 13.5 to 57 ng/mL extending over Days 11 to 60 in individual females. After peripheral P_4_ peaked, a gradual decline began, usually at Day 44 in most of the queens that were examined. The P_4_ levels were maintained at a relatively constant level until a few days before parturition, when they sharply decreased [37]. In the present study, serum P_4_ decreasing to values lower than 10 ng/mL (range 3.8 – 9.2 ng/mL) was observed in queens in the 7^th^ and 8^th^ week of pregnancy; however, in three individuals at the 6^th^ and 7^th^ week of pregnancy, the P_4_ values were still near 30 ng/mL. Summarizing these data, it might be concluded that, besides visible differences existing in plasma P_4_ levels between individuals, the distinct decrease in peripheral P_4_ precedes onset of parturition.

The successful extraction of P_4_ from the placenta further supports its role as an additional source of P_4_ during pregnancy in the cat. Placental P_4_ concentrations seem to be dependent on gestational age. Thus, the higher P_4_ values reported for pregnant queens may result from P_4_ supplementation by placental tissue, as hypothesized previously [18]. Still remaining, however, is the question whether the placenta-originating P_4_ would be alone able to maintain pregnancy in domestic cats ovariectomized after 45 days of pregnancy. In contrast to cats, in mice it was proven that ovariectomy at any time of pregnancy causes abortion [38]. It was thought that P_4_ synthesis is not possible *de novo* in the rodent placenta. Nevertheless, trophoblast giant cells from mouse placenta were shown to produce P_4_ from cholesterol [39]. Moreover, recent studies using molecular tools have shown that P_4_ synthesis is possible in rodent maternal decidual cells and occurs upon decidualization, but is terminated at mid-gestation [40]. It is further assumed that this local synthesis of P_4_ may act as an immunosuppressive factor at the implantation sites [41]. In contrast in cows placenta-derived progesterone and oestrone are supposed to be auto- or paracrine factors involved in placental growth and differentiation [42]. The synthesis of P_4_ was recently demonstrated in uninucleate trophoblast cells and the trophoblast giant cells of the bovine placenta; however, these two kinds of cells were reported to have different steroidogenic capacities [43].

The morphological comparative study in the epitheliochorial and endotheliochorial placenta types published by Leiser and coworkers in 1998 [44] presents a photograph of the typical labyrinthine-like system in the cat placenta with very easily visible and differentiated decidual cells [44]. In the present study, anti-vimentin staining was applied in order to distinguish between cells of mesenchymal origin and other sources, as described and confirmed for the canine and feline placenta by Bezler [34]. Since the StAR- and 3βHSD-positive placental cells were identified as maternal decidual cells, and were found mostly in placentas from the second half of gestation, the present findings on the spatio-temporal expression of the key steroidogenic genes differ from data obtained for other species. Even though some 3βHSD-positive cells were observed in the trophoblast, the strongest signals were present in decidual cells suggesting that these cells may be the main source of steroid synthesis within the feline placenta. Although the reproductive biology characteristics of the dog and cat are often compared, especially on this point distinct differences are observed, because in dogs the CL is the only source of P_4_ during both pseudopregnancy and pregnancy [7].

## Conclusions

The cellular localization of the steroidogenic factors that are present in the feline placenta differs from their localizations in rodents and cows. The present data confirm previous observations that the feline placenta is a supplemental source of P_4_ and could be important for the maintenance of pregnancy in this species.

## Competing interests

The authors declare that they have no competing interests.

## Authors’ contributions

MJS conceived of the study, and participated in its design, carried out molecular genetic studies and drafted the manuscript. EJ and AZS carried out the immunoassays and participated in molecular studies. AB and DJS helped to draft the manuscript. MPK participated in the design of the study and interpretation of the results, carried out the molecular and genetic studies, provided the knowledge- and methods-transfer and helped to draft the manuscript. The study was partially financed from the Institute of Veterinary Anatomy, University of Zurich, Zurich, Switzerland. All authors read and approved the final 552 manuscript.
